# Gender Disparities in Prolactinomas: Unravelling Clinical Patterns, Metabolic Variations, and Treatment Responses

**DOI:** 10.7759/cureus.42911

**Published:** 2023-08-03

**Authors:** Mohammad Salem Baba, Sajad Ul Islam Mir, Moomin Hussain Bhat, Bashir Ahmad Laway, Raiz Ahmad Misgar

**Affiliations:** 1 Endocrinology, Government Superspeciality Hospital, Srinagar, IND; 2 Endocrinology, Hamdard Institute of Medical Sciences and Research, New Delhi, IND; 3 Endocrinology, Sher-I-Kashmir Institute of Medical Sciences, Srinagar, IND

**Keywords:** prolactin levels, pituitary gland, metabolic syndrome, hyperprolactinemia, prolactinoma

## Abstract

Background and objective

Individuals with prolactinoma exhibit elevated rates of obesity, metabolic syndrome (MS), and dyslipidemia compared to their healthy counterparts. However, there is a lack of data regarding metabolic variance between male and female prolactinoma patients. Consequently, this study aimed to investigate and compare sex-specific discrepancies in metabolic abnormalities among individuals diagnosed with prolactinoma.

Methods

In this prospective study, 80 treatment-naïve patients with prolactinoma (12 males and 68 females) underwent clinical assessments and laboratory investigations. The measured parameters included blood glucose, total cholesterol (TC), triglycerides (TG), LDL cholesterol (LDL-C), HDL cholesterol (HDL-C), urea, creatinine, uric acid, and blood glucose levels. The patients were treated with cabergoline, a dopamine agonist, and reevaluated after 12 weeks.

Results

Forty-eight patients had microprolactinomas (all females), and 32 had macroprolactinomas (20 females, 12 males). The mean age was 28.30±7.49 years for females and 28.91±7.12 years for males (p=0.71). The median symptom duration was 12 months (range 1-72 months, IQR 4-16 months), with no significant difference between males (median 12 months, IQR 5-54 months) and females (median 12 months, IQR 10-24 months, p=0.620). The median serum prolactin (PRL) was 988 ng/mL (IQR 471-1,439) in males and 165 ng/mL (IQR 90-425) in females (p<0.05). Males showed higher HbA1c, BGF, TC, TG, LDL-C, and higher rates of obesity, MS, and diabetes mellitus. Treatment with cabergoline resulted in significant improvements in the HbA1c, BGF, TC, TG, and LDL-C levels.

Conclusion

Males with prolactinomas had larger tumor sizes and higher serum PRL levels than females. Additionally, males exhibited worse metabolic parameters than females. However, there was no significant difference in the duration of symptoms or age at diagnosis between the two groups.

## Introduction

Prolactinomas, the most common tumors of the pituitary gland and a leading cause of hyperprolactinemia (HPL), exhibit distinct sex-based characteristics [1,2]. These tumors are more frequently observed in females, with the majority being smaller than 1 cm in size. In contrast, males tend to present with larger prolactinomas and are typically older at the time of diagnosis [3-5]. The clinical manifestations of prolactinomas can be attributed to either HPL, resulting in symptoms such as galactorrhea, menstrual irregularities, infertility, decreased libido, or mass effects, causing headaches, visual disturbances, or, in rare cases, cranial nerve palsy [6,7]. HPL, in addition to its impact on the breast and reproductive organs, is associated with systemic effects such as weight gain, metabolic syndrome (MS), insulin resistance (IR), and chronic low-grade inflammation [8-11]. The primary treatment approach for prolactinomas involves the use of dopamine agonists (DA), while surgery and radiotherapy are typically reserved for cases involving large, drug-resistant, or recurrent tumors [12-15].

It is worth noting that the clustering of these risk factors may predispose patients with prolactinomas, especially males [16], to cardiovascular diseases. Despite previous studies highlighting the disparity in tumor size and serum PRL levels between males and females, limited data exist regarding differences in metabolic abnormalities between the two groups [17-20]. In an effort to contribute to existing knowledge, this study aimed to investigate sex differences in the clinical profile, metabolic abnormalities, and treatment outcomes of patients with prolactinomas.

## Materials and methods

Subject selection

This prospective cohort study was conducted in the endocrinology department of a tertiary care hospital between October 2018 and January 2023. The study enrolled patients diagnosed with prolactinoma who met specific criteria: signs and symptoms of HPL, serum PRL levels above the upper limit of normal, and the presence of an adenoma on pituitary magnetic resonance imaging (MRI). Patients taking medications known to cause HPL and those with polycystic ovary syndrome (PCOS), chronic liver disease, or chronic kidney disease were excluded. Written informed consent was obtained from all participants and the study protocol was approved by the institution's ethics committee.

Clinical assessment

All study participants underwent comprehensive clinical assessment. Detailed medical histories were obtained, with a particular focus on menstrual disturbances (amenorrhea and oligomenorrhea) in women, premature ejaculation or erectile dysfunction in men, galactorrhea, infertility, decreased libido, weight gain, headache, visual disturbances, and medication use. Blood pressure (BP) measurements and anthropometric assessments were performed, including height, weight, waist circumference (WC), and hip circumference (HC). These measurements were performed by a single examiner, with the participants barefoot and wearing light clothing. Height was measured using a wall-mounted stadiometer (SECA 13, Hamburg, Germany), whereas body weight was measured using a digital scale balance (SECA 13, Hamburg, Germany). WC was measured at the midpoint between the lowest rib margin and the iliac crest, and HC was measured at the widest level over the greater trochanters. Body mass index (BMI) was classified as overweight, those with a BMI of 23-27.4 kg/m^2^ were classified as overweight, and those with a BMI of 27.5 kg/m^2^ or higher were classified as obese [21]. The International Diabetes Federation (IDF) criteria were used to define MS. According to the IDF criteria, MS is characterized by the presence of central obesity (WC > 90 cm in males and >80 cm in females), along with any two of the following: hypertension (≥130/85 mm Hg), fasting plasma glucose (FPG) levels ≥100 mg/dL, triglyceride (TG) levels ≥150 mg/dL, or low high-density lipoprotein (HDL) cholesterol levels (≤50 mg/dL in females and <40 mg/dL in males) [22]. In men, central hypogonadism was defined as the presence of suggestive signs and symptoms, with total serum testosterone levels below 250 ng/dL and luteinizing hormone (LH) levels below 10 IU/L. In women, central hypogonadism was defined as cessation of menstrual cycles for more than three months and follicle-stimulating hormone (FSH) levels below 5 IU/L [23].

Laboratory measurements

A baseline early morning fasting blood sample was drawn from all patients and controls for the following investigations: hemoglobin (Hb), total leukocyte count(TLC), platelet counts, urea, creatinine, bilirubin, alanine aminotransferase (ALT), alkaline transferase (ALP), total protein, albumin, glucose, glycated hemoglobin (HbA1c), total cholesterol (TC), high-density lipoprotein (HDL) cholesterol, low-density lipoprotein (LDL) cholesterol, triglycerides (TG), calcium (Ca), phosphorous (PO_4_), uric acid (UA), triiodothyronine (T3), tetra iodothyronine (T4), thyroid-stimulating hormone (TSH), PRL, FSH, LH, total testosterone, cortisol, ESR, hsCRP, ICAM-1, and VCAM-1. Blood was allowed to clot at room temperature (15-25 degree Celsius) and centrifuged for 15 min to obtain hemolysis-free serum. The serum was collected in separate plastic tubes, and some parts were stored at -70 degree Celsius until further analysis.

Measurements of urea, creatinine, bilirubin, ALT, ALP, total protein, and albumin were performed using the same-day automated chemistry analyzer (HITACHI-912). Plasma glucose was estimated on the same day by an enzymatic method using glucose oxidase and peroxidase on an automated chemistry analyzer (HITACHI-912). Lipid parameters were analyzed on the same day using commercially available enzymatic reagents (Audit Diagnostics, Ireland) adapted to the HITACHI-912 autoanalyzer. HbA1c was measured using high-performance liquid chromatography standardized to the Diabetes Control and Complications Trial (DCCT) assay on an Avantor A9 HbA1c analyzer with whole blood collected in ethylenediaminetetraacetic acid (EDTA) tubes. Urea, creatinine, bilirubin, ALT, ALP, total protein, albumin, glucose, HbA1c, lipid profile, Ca, PO4, and uric acid levels were estimated on the same day. Serum PRL (normal range:1-27 µg/L (women); 1-20 µg/L (men)), TSH, T3, T4, FSH, LH, cortisol, and testosterone were measured using a commercial Chemiluminescent Immunoassay (Beckman Coulter Unicel, DXI).

Imaging

All patients enrolled in the study underwent contrast-enhanced MRI of the sellar, parasellar, and suprasellar regions using a 1.5-tesla MRI scanner (Siemens, Magneton Avanto, Germany). The MRI protocol included pre-contrast T1- and T2-weighted spin-echo coronal and sagittal sections with the following specific imaging parameters: a small field of vision (20×25 cm), thin slices (3 mm), and a high-resolution matrix (256×512). To detect small adenomas, dynamic contrast studies were performed following bolus injection of intravenous gadolinium. Six consecutive sets of three images were acquired in the coronal plane, and each set was captured every 10 s. This dynamic imaging technique enhances the visibility of small adenomas. Adenomas were classified as microprolactinomas if they measured less than 10 mm in size, while those equal to or larger than 10 mm were categorized as macroprolactinomas [24]. For patients diagnosed with macroprolactinomas, perimetry was performed using a Humphrey field analyzer to assess the visual field defects.

Statistical analysis

Statistical software used for data analysis was Statistical Package for Social Sciences (SPSS) version 20 (IBM Corp., Armonk, NY). The Kolmogorov-Smirnov test was used to assess the normality of the samples. Descriptive statistics, such as mean and standard deviation, were used to present continuous variables that followed a normal distribution, whereas median and interquartile range were used for non-normally distributed data. Categorical variables are described as frequencies and percentages. The chi-square test was used to compare the categorical variables. Student's independent t-test and Mann-Whitney U test were used for normally and non-normally distributed continuous variables, respectively. In cases where it was necessary, data were log-transformed. Partial correlation and regression analyses were performed, as described in the text. All results were considered significant at the 5% significance level (p<0.05).

## Results

Eighty consecutive patients (12 men and 68 women) were enrolled in the study. Among them, 48 patients had microprolactinomas (all females), while 32 patients had macroprolactinomas (20 females and 12 males). The mean age of females was 28.30±7.49 years, and for males, it was 28.91±7.12 years (p=0.71). The median duration of symptoms was 12 months, ranging from 1 to 72 months, with an interquartile range (IQR) of 4-16 months. There was no significant difference in the duration of symptoms between macroprolactinomas (median, 12 months, IQR 12-30 months) and microprolactinomas (median, 12 months, IQR 6-24 months, p=0.24). Similarly, no significant difference was observed in the duration of symptoms between males (median 12 months, IQR 5-54 months) and females (median 12 months, IQR 10-24 months, p=0.620).

In females, the most common symptom was galactorrhea (75%), followed by headache (55%), oligomenorrhea (40%), infertility (38%), and secondary amenorrhea (30%). In males, the most common symptom was headache (90%), followed by visual field abnormalities (77%) (Table 1). Approximately 32% of females and 56% of males were classified as obese, whereas MS was diagnosed in 78% of males and 47% of females. New-onset diabetes mellitus (DM) was detected in 4% of females and 23% of males. Additionally, 6% of females and 22% of males had hypertension. Overall, 45% of males and 15% of females exhibited central hypogonadism (Table 2). The median maximum tumor diameter was 8.5 mm (IQR 6-13.3 mm) in females and 29 mm (IQR 24.5-36.5 mm) in males. The perimetry revealed abnormalities in 13 patients. One female patient was diagnosed with MEN1 syndrome.

**Table 1:** Symptoms of prolactinomas in males and females

Females (n=68)		Males (n=12)	
Symptom	Percent	Symptom	Percent
Galactorrhoea	75	Headache	90
Menstrual abnormality	71	Visual abnormalities	77
Headache	55	Weight gain	55
Infertility	38	Infertility	22
Weight gain	19	Erectile dysfunction	7
Visual abnormality	13		

**Table 2:** Demographic and anthropometric parameters of female and male prolactinoma patients Values expressed as mean ±SD unless specified, # expressed as median and IQR SD: standard deviation; IQR: interquartile range; BMI: Body Mass Index; WC: Waist Circumference; WHR: Waist-Hip Ratio; HbA1C: Glycated Hemoglobin; BGF: Blood Glucose Fasting; TC: Total Cholesterol; TG: Triglycerides; LDL-C: LDL cholesterol; HDL-C: HDL cholesterol; PRL: Prolactin; T3: Triiodothyronine; T4: Tetra iodothyronine; TSH: Thyroid-stimulating hormone; FSH: Follicle-stimulating hormone; LH: Luteinizing hormone

Parameter	Females (n=68)	Males (n=12)	P-value
Age (years)	28.30±7.90	28.91±7.26	0.708
Weight (kg)	65.33±14.93	84.84±11.37	0.005
BMI (kg/m^2^)	25.49±5.23	28.58±3.97	0.059
WC (cms)	87.89±13.51	94.45±9.52	0.049
WHR	0.931	0.987	0.058
Maximum adenoma diameter^#^ (mm)	8.5(6-13)	29(24-36)	<0.01
Duration of symptoms (months)^#^	12(9-24)	12(5-54)	0.620
Hypertension	6%	22%	0.139
Diabetes mellitus	4%	23%	0.054
Metabolic syndrome	47%	78%	0.041
Hypogonadism	15%	45%	0.041
Obese	32%	56 %	0.256
HbA1C (%)	5.35±0.99	6.04±0.47	0.001
BGF (mg/dL)	95.87±29.09	105.94±10.51	0.011
TC (mg/dL)	166.91±39.70	205.01±39.87	0.002
TG (mg/dl)	151.10±57.67	199.73±53.90	0.015
LDL-C (mg/dl)	108.19±28.04	133.64±24.81	0.003
HDL-C (mg/dl)	41.01±6.69	37.67±9.50	0.674
PRL^#^(ng/ml)	156 (90-425)	988(471-1539)	<0.01
T3 (ng/ml)	1.28±0.41	1.27±0.37	0.886
T4 (ug/dl)	8.45±1.60	7.27±1.04	0.883
TSH (uIU/ml)	3.59±2.18	3.90±2.05	0.721
FSH (IU/L)	7.17±3.14	2.74±1.35	0.003
LH (IU/L)	5.08±2.08	2.41±1.32	0.005
Cortisol (ug/dl)	12.45±3.31	10.90±2.42	0.462

The median serum PRL levels in males and females were 988 ng/mL (IQR 471-1439) and 165 ng/mL (IQR 90-425), respectively (p<0.05). Males exhibited lower mean levels of FSH (7.17±3.14 vs 2.74±1.35 IU/L, p=0.003) and LH (5.08±2.08 vs 2.41±1.32 IU/L, p=0.005) compared to females. Conversely, males demonstrated higher mean levels of HbA1c (5.35±0.99 vs 6.04±0.47%, p=0.001), BGF (95.87±29.09 vs 105.94±10.51 mg/dL, p=0.011), TC (166.91±39.70 vs 205.01±39.87 mg/dL, p=0.002), TG (151.10±57.67 vs 199.73±53.90 mg/dL, p=0.015), and LDL-C (108.19±28.04 vs 133.64±24.81, p=0.003) (Table 2). In the partial correlation analysis, controlling for age and weight, serum PRL levels exhibited a strong positive correlation with the maximum adenoma diameter (r=0.768, p<0.001). Additionally, serum PRL levels were weakly correlated with TC (r=0.245, p=0.014), LDL (r=0.276, p=0.006), and uric acid (UA) (r=0.239, p=0.017) (Table 3).

**Table 3:** Correlation⁺ between PRL© and various parameters (controlled for age and weight) # Spearman correlation coefficient, ⁺partial correlation analysis, © log 10 of PRL UA: Uric Acid; HbA1C: Glycated Hemoglobin; BGF: Blood Glucose Fasting; TC: Total Cholesterol; TG: Triglycerides; LDL-C: LDL cholesterol; HDL-C: HDL cholesterol

Parameter	r^#^	P-value
Duration of symptoms	0.047	0.745
Maximum adenoma size	0.768	<0.001
UA	0.239	0.017
HbA1C	0.033	0.815
BGF	0.144	0.155
TC	0.245	0.014
TG	0.186	0.066
LDL-C	0.276	0.006
HDL-C	0.010	0.918

A total of 56 patients (47 females and nine males) underwent reassessment three months after cabergoline treatment. Among them, 49 (44 females and five males) achieved normal serum PRL levels. In six patients (two females and four males), PRL levels decreased by more than 50%, and in one female patient, the decrease in serum PRL levels was less than 50%. Repeat MRI scans at three months were performed in 20 patients, with 60% (eight females and four males) showing a > 50% reduction in adenoma size, and 40% (four females and four males) exhibiting a less than 50% decrease in adenoma size. At 12 weeks of follow-up, significant improvements were observed in various metabolic parameters. The mean weight of patients decreased from 70.428±14.93 kg to 67.28±13.51 kg (p<0.001), BMI decreased from 26.49±5.23 kg/m^2^ to 25.70±5.11 kg/m^2^ (p=0.001), and WC decreased from 89.89±13.51 cm to 87.06±12.10 cm (p<0.001). Furthermore, there was a notable improvement in metabolic parameters such as HbA1c (5.45±0.99% vs 5.08±0.49%, p<0.001), BGF (96.87±29.09 vs 86.30±9.73 mg/dL, p=0.003), TC (172.91±39.70 vs 147.89±42.71 mg/dL, p<0.001), TG (159.10±57.67 vs 141.36±51.48 mg/dL, p=0.025), and LDL-C (112.19±28.04 vs 101.81±26.10 mg/dL, p=0.008) after treatment with cabergoline. However, HDL-C (41.01±6.69 vs 41.34±5.03 mg/dL, p=0.333) did not exhibit a significant change (Table 4).

**Table 4:** Baseline and follow-up parameters of patients after 12 weeks of treatment (n=56) Values expressed as mean ± SD unless specified SD: Standard Deviation; BMI: Body Mass Index; WC: Waist Circumference; WHR: Waist-Hip Ratio; HbA1C: Glycated Hemoglobin; BGF: Blood Glucose Fasting; TC: Total Cholesterol; TG: Triglycerides; LDL-C: LDL cholesterol; HDL-C: HDL cholesterol

Parameter	Baseline	Follow up	P-value
Weight (kg)	70.42 ±14.93	67.28±13.51	<0.001
BMI (kg/m^2^)	26.49±5.23	25.70±5.11	0.001
WC (cm)	89.89±13.51	87.06±12.10	<0.001
WHR	0.97±0.08	0.95±0.08	0.006
HbA1c (%)	5.45±0.99	5.08±0.49	<0.001
BGF (mg/dL)	96.87±29.09	86.30±9.73	0.003
UA (mg/dL)	4.83±1.31	3.90±1.11	0.011
TC (mg/dL)	172.91±39.70	147.89±42.71	<0.001
TG (mg/dL)	159.10±57.67	141.36±51.48	0.025
LDL-C (mg/dL)	112.19±28.04	101.81±26.10	0.008
HDL-C (mg/dL)	41.01±6.69	41.34±5.03	0.333

## Discussion

This study, conducted in the Indian subcontinent, provides significant insights into the clinical and metabolic differences between male and female patients with prolactinoma. As previously documented in multiple studies, females outnumbered males in our cohort [14,17,25]. Notably, the majority of tumors in females were microprolactinomas, while all males in this study presented with macroprolactinomas, consistent with findings from the existing literature [7,17,26-28]. However, it should be noted that some authors, primarily from Asia, have reported equal prevalence of macroprolactinomas and microprolactinomas in men [29,30]. Although it is commonly reported that most prolactinomas in men are diagnosed around the fifth decade of life [17], the median age of presentation in both males and females in our study was approximately 28 years, consistent with findings from other studies [26,27,29,31]. However, it is important to note that the small sample size of this study may not have provided accurate estimates of age differences. Furthermore, we observed no significant differences in age at diagnosis or duration of symptoms between males and females. The larger tumors in males with higher prolactin levels suggest that the size discrepancy in tumors between sexes is not primarily due to a diagnostic delay, but rather reflects gender-related differences in tumor behavior, potentially involving the estrogen receptor pathway [27,31].

In terms of metabolic abnormalities, it is well established that patients with prolactinomas have higher weight, fat content, IR, and lipid levels than their healthy counterparts. However, it remains unclear whether these metabolic differences are sex-specific [32,33]. In our study, we found that males had worse metabolic abnormalities than females, including higher BMI, blood glucose levels, and lipid levels, as well as an increased prevalence of obesity, MS, hypertension, and diabetes. The clustering of these risk factors may contribute to an elevated risk of cardiovascular disease, particularly in males with HPL [16,34]. Various theories have been proposed to explain the weight gain observed in HPL, including decreased dopaminergic tone, increased adiposity, and altered leptin and adiponectin responses [33,35-37].

Notably, the common presenting features in females were galactorrhea and menstrual cycle abnormalities, whereas mass effects such as headache and visual field abnormalities were the most common features in males, which is consistent with previous data [7,26,38].

Following a 12-week treatment with cabergoline, approximately 88% of the patients achieved normal serum PRL levels, consistent with the findings of previous studies [3,14,25,26]. Similarly, approximately 60% of the patients experienced a greater than 50% decrease in adenoma size, while 40% exhibited a less than 50% decrease. A retrospective study by Berinder et al. reported a 71% rate of serum PRL level normalization and 80% showed either total or partial tumor shrinkage [26]. In the present study, significant improvements in weight and lipid and HbA1c levels were observed after a 12-week follow-up period. These findings align with the similar improvements in metabolic parameters reported by Berinder et al. [39] and Pala et al. [40]. Furthermore, Santos-Silva et al. reported that after six months of treatment with DA, serum PRL levels normalized, although no significant difference in BMI was observed. However, this study demonstrated a noteworthy decrease in the IR index, glucose levels, LDL cholesterol, and triglyceride levels [9].

Limitations

While this study provides valuable insights into the clinical and metabolic differences between male and female prolactinoma patients, there are some limitations that should be acknowledged. First, the sample size was relatively small, which may limit the generalizability of the findings to a larger population. Furthermore, the study primarily relied on clinical and laboratory data, without considering other potential factors that may influence metabolic parameters, such as lifestyle, diet, and physical activity. Incorporating these factors into the analysis would provide a more comprehensive understanding of observed metabolic differences. Lastly, the study did not explore the underlying mechanisms behind the observed sex differences in tumor behavior and metabolic abnormalities. Future research should investigate the specific pathways and hormonal influences that contribute to these disparities.

## Conclusions

Most prolactinoma patients were females. Male prolactinoma patients tend to have larger tumors and higher serum PRL levels than female patients. Additionally, males exhibited worse metabolic parameters than females. However, there were no significant differences in the duration of symptoms or age at diagnosis between the two sexes.
